# A novel immunomodulatory and molecularly targeted strategy for refractory Hodgkin's lymphoma

**DOI:** 10.18632/oncotarget.1468

**Published:** 2013-11-19

**Authors:** Vivek Subbiah, Robert E. Brown, Mary F. McGuire, Jamie Buryanek, Filip Janku, Anas Younes, David Hong

**Affiliations:** ^1^ Department of Investigational Cancer Therapeutics (Phase I Clinical Trials Program), Division of Cancer Medicine, The University of Texas MD Anderson Cancer Center, Houston, TX; ^2^ Department of Pathology & Laboratory Medicine, UT Health, University of Texas-Houston Medical School, Houston, Texas,; ^3^ Memorial Sloan-Kettering Cancer Center, Memorial Hospital, New York, NY

**Keywords:** Hodgkin's lymphoma, PI3K/AKT/mTOR, mTOR, HDAC, immune dysregulation, morphoproteomics, biomedical analytics, vorinostat, sirolimus, adolescent and young adult oncology, rapalog, targeted therapy, immunotherapy, unusual responder, exceptional responder, complete response, FDG-PET, brentuximab vedotin, CD8, CD30, T-cell regulatory cells

## Abstract

Although Hodgkin's lymphoma (HL) was one of the first human cancers to be cured by chemotherapy, no new agents other than brentuximab vedotin (Adcetris®, CD 30 directed antibody drug conjugate) have received US Food and Drug Administration (FDA) approval for HL since 1977. Subsets of young adult patients with HL continue to relapse, even after stem cell transplantation, warranting new approaches. Against this background, we report a dramatic response in a young patient with advanced HL refractory to the standard treatment who responded to the combination of a pan-histone deacetylase inhibitor (vorinostat, suberoylanilide hydroxamic acid, SAHA) and mammalian target of rapamycin (mTOR) inhibitor therapy (sirolimus,rapamume). In-depth immunohistochemical and morphoproteomic analyses of this exceptional responder to targeted therapy have yielded potential insights into the biology of advanced HL. The PI3K/AKT/mTOR pathway is a commonly activated pathway in multiple tumor types including HL. The patient was treated using therapy based on mechanistic in vitro data demonstrating that combined histone deacetylase (HDAC) and mTOR inhibition act together on this pathway, resulting in inhibition of reciprocal feedback networks, leading to better anti-proliferative activity. The in vivo response signature from this patient's tissue sample sheds light on immune dysregulation in HL. We describe the response signature achieved from targeting immune dysregulation in addition to targeting the key oncogenic PI3K/AKT/mTOR pathway. We also expand on the role of rapamycin analogs in oncology. This study supports a role for an immune-type pathogenesis that is amenable to immune modulating targeted therapy in refractory HL.

Significance: We report an exceptional responder to molecularly targeted and immune modulator therapy in advanced Hodgkin's lymphoma. The morphoproteomic/morphometric findings in this “unusual responder” patient's relapsed HL that correlate best, as a response signature with the subsequent clinical remission following rapamycin (sirolimus) and vorinostat (SAHA) therapies, center on an immune dysregulation involving an imbalance between effector and functional T regulatory cells in addition to targeting the mTOR pathway. This underscores the need for an approach illustrated in our study – namely of focusing on pathogenetic mechanisms and combinatorial therapies that target both the pathogenesis and adaptive responses to contemplated therapies.

## INTRODUCTION

Originally described by Thomas Hodgkin in 1832, and described as “morbid experiences of the absorbent glands and spleen”, Hodgkin's lymphoma (HL) continues to be an intriguing, and fascinating lymphoma[1]. Over time, HL became designated as a lymphoma characterized by atypical and distinctive Reed-Stenberg and Hodgkin (R-S/H) cells[2], justifying the use of cytotoxic chemoradiation therapies[2-6]. The documentation of clonality in the R-S/H cells, albeit in fewer than 50% of cases of classic HL, has reinforced the school of thought that HL is indeed a lymphoma[2-5, 7]. However, the absence of clonal evolution in most HL cases suggests that other factors may be responsible for its development and progression[8]. In short, a common pathogenetic sequence may be operative in the development of both the lymphoma and the accompanying clonal evolution, when present. Immune dysregulation, specifcally autoimmunity, is a commonality that might explain the pathogenesis of HL in addition to overactive key oncogenic signaling pathways such as the mammalian target of rapamycin (mTOR) pathway.

Against this background, we report the case of a patient with advanced Hodgkin's lymphoma refractory to the standard treatment who eventually responded to the combination of a pan-deacetylase inhibitor (vorinostat, suberoylanilide hydroxamic acid ,SAHA) and mTOR inhibitor therapy (sirolimus, rapamune®). Occasionally, in oncology an index patient has a dramatic and uncommon response to therapy. Much can be learned by intensively studying the underlying mechanisms of response in such a patient with the hopes of gaining new insights that will lead to benefcial new therapies for refractory lymphoma. We hypothesized that performing molecular analysis to explicate the response signature in this “*exceptional responder*” would help shed light on the biology of aggressive Hodgkin's lymphoma and instruct and possibly predict future patients with this lymphoma. We present molecular and biomedical analytic evidence in support of the efficacy of immune modulating therapies such as the one used to treat our patient in addition to targeting oncogenic pathways in the successful treatment of HL. Our findings reinforce the concept of immune dysregulation in the pathogenesis of classic HL.

## RESULTS

### Patient clinical course and treatment history

**Figure 1:**
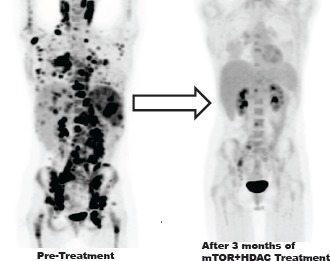
FDG PET/CT scans showing metabolic response to therapy Response in the 26 year old female with classic Hodgkin's lymphoma with sirolimus and vorinostat after 6 lines of therapy. Pre-treatment scans show extensive lymphoma. Scans after 3 cycles of therapy show an exceptional response and decreased SUV activity.

A 26-year-old white female was originally diagnosed with stage III A Hodgkin's lymphoma. Histopathologically, her tumor was classical nodular sclerosis Hodgkin's lymphoma. She started therapy with standard of care ABVD chemotherapy (doxorubicin, bleomycin, vinblastine and dacarbazine) for 6 cycles. Unfortunately, she had persistent lymphoma. She then received ifosfamide, carboplatin and etoposide (ICE) therapy for 2 cycles with residual lymphoma. This was followed by high-dose BCNU, etoposide, cytosine arabinoside, and melphalan (BEAM) followed by autologous stem cell transplantation. After transplantation, the patient had persistent lymphoma in the left supraclavicular area and received radiation to the left neck with a total dose of 43.6 Gy. She was subsequently enrolled on an anti-CD30-brentuximab vedotin study (SGN-35) for 5 cycles. Unfortunately, she developed progressive lymphoma, which was followed by a double cord blood transplant. Her lymphoma continued to progress. She was then started on lenalidomide with the addition of anti-CD 20 monoclonal antibody (rituximab). Following progression, she was enrolled on protocol treatment with an AKT inhibitor, MK2206, (NCT01258998) with progression of lymphoma after 2 cycles and then received 2 cycles on a STAT3 inhibitor trial (NCT01563302). Positron emission tomography and computed tomography (PET/CT) scans were done (Figure 1 A), which showed increasing lymphoma involving lymph nodes, liver, spleen and bones. Unfortunately, she was not eligible for any clinical trials due to her abnormal blood counts. Given the patient's good performance status, young age and motivation, she was treated with vorinostat, a pan-deacetylase inhibitor combined with sirolimus, an mTOR inhibitor. Other than grade 3 thrombocytopenia requiring platelets, she tolerated therapy reasonably well. A restaging PET/CT after 3 cycles of therapy noted a dramatic metabolic response to therapy (Figure 1 B). Throughout the course of this therapy, her Eastern Cooperative Oncology Group performance status (ECOG PS) remained at zero. Following combination therapy with sirolimus (rapamycin) and vorinostat, the patient underwent a HLA-matched related donor transplant and remains free of lymphoma. She continued on rapamycin and vorinostat after the transplant with no evidence of lymphoma since initiation of this treatment for a total of more than 14 months from the time of this report. After obtaining informed consent from the patient, we performed morphoproteomic and biomedical analytics incorporating Ingenuity Pathway Analysis (IPA) to delineate response signatures.

### Molecular analyses

**Figure 2:**
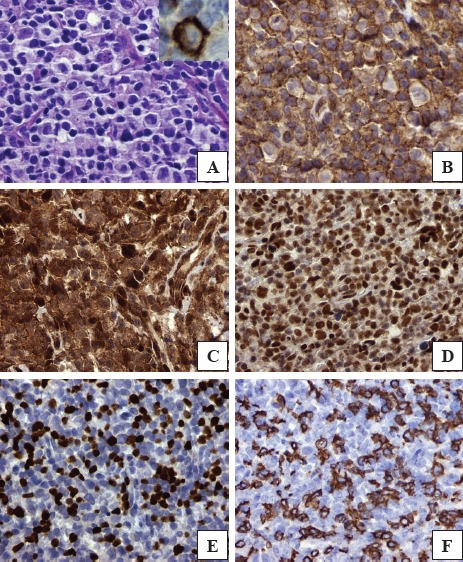
Patient biopsy specimen containing lesional R-S/H cells and companionate lymphocytes H&E stained section (Frame A; inset shows CD30 positivity); plasmalemmal expression of insulin-like growth factor (IGF)-1 receptor[Tyr1165/1166] (Frame B); p-Akt [Ser 473] detected on the plasmalemmal-cytoplasmic aspect and in occasional nuclei (Frame C); Sirt1 expression in the majority of the nuclei (Frame D); Foxp3 showing nuclear expression in companionate lymphocytes (Frame E); and CD8 expression(Frame F) on the plasmalemmal aspect of companionate lymphocytes (TIA-1 and p-mTOR [Ser 2448], not depicted; DAB brown chromogenic signal; original magnifications x600 for Frames A and B and x400 for Frames C-F).

The hematoxylin-eosin stained section of the patient's HL revealed R-S/H cells and companionate lymphocytes (Figure 2, Frame A). Morphoproteomic analysis[9] demonstrated the following: CD30 immunopositivity on the plasmalemmal aspect of R-S/H cells (Figure 2, Frame A with inset); the insulinlike growth factor(IGF) pathway as evidenced by the expression (up to 2+ on a scale of 0 to 3+) of IGF-1R (Tyr1165/1166) on the plasmalemmal aspect of R-S/H cells and companionate lymphocytes (Figure 2, Frame B); correlative expression and activation of the downstream pathway of convergence in IGF signaling in the form of mTOR, phosphorylated on serine 2448 with up to 3+ nuclear and 1+ cytoplasmic expression scores and p-Akt (Ser 473) at up to 1+ in the cytoplasmic-plasmalemmal compartment and occasionally up to 3+ in the nuclear compartment, supporting activation of the mTORC2 pathway (see Figure 2, Frame C).Sirt 1, an NAD+ histone deacetylase and an immune modulating protein was noted up to 3+ in the nuclei of the R-S/H cells and in the vast majority of the companionate lymphocytes (Figure 2, FrameD). The numbers of Foxp3+, T regulatory cells and CD8+ and TIA-1+ effector cells were as many as 150, 130 and 70 cells, respectively, per high power field (Figure 2, Frames E, and F).

### Biomedical Analytics

**Figure 3:**
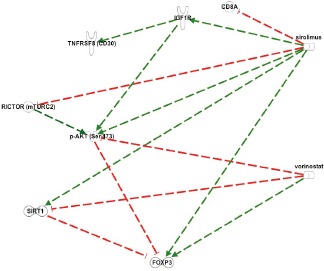
Key interactions modulated by sirolimus and vorinostat in Hodgkin's Lymphoma Dashed lines: indirect interactions. Red coloration/t-bar: downregulation: Green coloration/arrow: upregulation. The rationale for combinatorial therapy with vorinostat and sirolimus (rapamycin) is refected in the potential for both the blunting of adaptive responses of the lymphoma process to rapamycin alone and also the beneft of combinatorial therapy to effect functional and expanded T regulatory cells to address an immune dysregulation. For example, rapamycin can, in the short term, result in increased phosphorylative activation of p-Akt on serine 473 by removing the feedback inhibition of IGF-1R pathways signaling through mTORC2. This is countered by vorinostat through the activation of protein phosphatase (PP); the action of the latter leads to dephosphorylation of Akt, including on serine 473. Additionally, rapamycin has been shown to upregulate Sirt1 expression and activity and this would be moderated by the inhibitory effect of vorinostat on Sirt1 at both the genomic and functional level. Moreover, because the actions of rapamycin and vorinostat result in the expansion and function of Foxp3 T regulatory cells, their use in combination should converge on righting any immune dysregulation.

In order to focus on our attempt to increase T regulatory (Foxp3) function, the network model evoked from Ingenuity Pathway Analysis (IPA, www.ingenuity.com) and additional MEDLINE sources was edited to emphasize the companionate lymphocyte reaction in HL and not the R-S/H cells (see Figure 3). Dashed lines show the indirect interactions (intermediate interactions deleted), with red/t-bar for downregulation and green/arrow for upregulation. Sirolimus inhibits CD8A and expands FOXP3, countering immune dysregulation. It also downregulates RICTOR (mTORC2), the putative serine 473 kinase for Akt. The complementary effects of sirolimus and vorinostat can be clearly seen in both upregulating Foxp3 expression and/or stability, and vorinostat modulating adaptive responses that might be seen with sirolimus alone.

## DISCUSSION

In-depth immunohistochemical and morphoproteomic analyses of an exceptional responder to targeted therapy have yielded potential insights into the biology of advanced HL. The PI3K/AKT/mTOR pathway is a commonly activated pathway in multiple tumor types including HL[10]. The patient was treated using therapy based on mechanistic in vitro data demonstrating that combined histone deacetylase (HDAC) and mTOR inhibition act together on this pathway, resulting in inhibition of reciprocal feedback loops, leading to augmented anti-proliferative activity[10]. The in vivo response signature from this patient's tissue sample sheds light on immune dysregulation in HL.

Through IPA pathway analyses and additional data mining of the MEDLINE data base as depicted in Figure 3, sirolimus is shown to inhibit CD8A and expand FOXP3, countering immune dysregulation. The complementary effects of sirolimus and vorinostat can be clearly seen in both upregulating Foxp3 expression and/or stability, and vorinostat modulating adaptive responses that might be seen with sirolimus alone.

In addition, vorinostat is known to downregulate SIRT1 gene mRNA and Sirt1 protein level and function[11, 12]. In the context of the expression of Foxp3 in the tumor infiltrating lymphocytes, it is likely that the SIRT1 effects a reduction in the suppressive function of these T regulatory cells[13, 14] which is likely to be reversed by vorinostat[11, 12].

The premise that HL is pathogenetically linked to an immune dysregulation is suggested by the following: 1. The reported association of HL with autoimmune diseases[15]; and 2. The unfavorable prognostic implications of high numbers of CD8+ and/or TIA+ effector T cells versus the favorable implications of high Foxp3 T regulatory cell numbers in the companionate lymphocytes of classical HL[16, 17]. The dramatic and durable complete clinical response in this patient to combinatorial therapy with vorinostat and sirolimus , when viewed in the context of their immune modulating properties, coincides with the morphoproteomic findings in her tissue specimen in supporting an underlying immune dysregulation of an autoimmune type as a pathogenetic factor in the patient's recurrent and previously refractory lymphoma. Specifically, biomedical analytics applied to these observations and data in her HL support a role for sirolimus in downregulating CD8 effector cell function while upregulating the numbers and suppressive function of Foxp3 T regulatory cells. The latter might involve interaction or synergy with the latency associated peptide of transforming growth factor beta, which one of us (REB) had previously reported as being expressed in the R-S/H cells and companionate lymphocytes in classic HL[18-20]. Additionally, sirolimus, because of its ability to inhibit mTORC2 assembly, after long-term exposure may lead to the cytoplasmic translocation of the mTORC2 component, sin1, and result in reduced mTORC2 degradation of Foxp3, thereby increasing its functional activity. Furthermore, vorinostat acts to enhance Foxp3, T regulatory cell expression and function by: 1. Dephosphorylating Akt on serine 473, reducing the rate of degradation of Foxp3 effected by mTORC2/Akt (Ser 473)[21] and promoting stabilization of Foxp3 and the functionality of the T regulatory cells[22] ; 2. Inhibiting the SIRT1 pathway's deacetylation of Foxp3 by virtue of its known ability to downregulate SIRT1 mRNA[11] and to inhibit the deacetylase activity of SIRT1[12] and, as a result, to reduce the destabilizing role of SIRT1 on Foxp3 expression and function[13, 14]; and 3. Expanding the T regulatory cell, Foxp3 population [22].

We note that rapamycin and/or rapamycin analogs have been previously shown to have a role in the treatment of various malignancies. Sirolimus induces remission of post-transplant lymphoproliferative disorders[23, 24] and of recurrent Epstein Barr virus-associated multilocular leiomyosarcoma after cardiac transplantation[25]. Relatedly, rapamycin has been reported to induce rapid regression of lymphadenopathy in a child with autoimmune lymphoproliferative syndrome[26]. Finally, rapamycin (sirolimus) and analogs such as temsirolimus and everolimus, (specifically the latter), have been approved for the treatment of metastatic renal cell carcinoma(RCC)[27, 28]. However, resistance to these agents in metastatic RCC can develop in association with adaptive switching from mTORC1 to mTORC2 signaling pathways and with concurrent activation of STAT3 and extracellular-signal regulated kinase (ERK) prosurvival/antiapoptotic pathways[28, 29]. This has been shown in other tumors such as sarcomas as well[30-32]. Moreover, in the case of solid tumors not associated with immune dysregulation, antitumoral immune surveillance could be compromised using rapamycin as a therapy by virtue of its potential role in the expansion and function of T suppressor (regulatory) cells in the tumoral microenvironment. This underscores the need for an approach illustrated in our study – namely of focusing on pathogenetic mechanisms and combinatorial therapies that target both the pathogenesis and adaptive responses to contemplated therapies. In addition the role of rapalogs have expanded in oncology to anti-aging as well[33, 34]. In pre-clinical models rapalogs have been shown to prevent age-related weight gain, decrease rate of aging, increase lifespan, and suppress carcinogenesis in transgenic cancer-prone mice[33, 35-37].

One of the limitations of this report is that the prolonged response to sirolimus and vorinostat lasted about 3 months before the patient also underwent an allogeneic transplant. It is difficult to know whether the subsequent one year remission was due to the sirolimus plus vorinostat or the allo-transplant. However, the patient underwent several transplants before the treatment; the dramatic response to the sirolimus/vorinostat combination – even if for only 3 months – gave this patient a deep remission that enabled the next transplant. This benefit cannot be discounted.

In summary, the morphoproteomic/morphometric findings in this “unusual responder” patient's relapsed HL that correlate best – as a response signature with the subsequent clinical remission following rapamycin (sirolimus) and vorinostat (SAHA) therapies – center on an immune dysregulation involving an imbalance between effector and functional T regulatory cells in addition to targeting the mTOR pathway. Morphoproteomic and biomedical analytics provide a response signature in the context of immune modulation, raising the question of immune dysregulation of the autoimmune type in HL. This supports the phase 1B clinical trial with combinatorial vorinostat and sirolimus from the immune modulating perspective, which is IRB approved and actively recruiting patients (NCT01266057). Preliminary results demonstrate a progress in the management of Hodgkin's lymphoma, even for heavily pre-treated patients.

## MATERIALS AND METHODS

We reviewed the medical record of a patient with HL who presented to the Department of Investigational Cancer Therapeutics at the The University of Texas MD Anderson Cancer Center seeking treatment options. Treatments, attaining informed consent, data collection, and molecular analysis were performed in accordance with the guidelines of The University of Texas MD Anderson Cancer Center Institutional Review Board (IRB). Molecular analyses and biomedical analytics were performed (RE Brown's Consultative Proteomics Laboratory) to analyze response signatures.

### Immunohistochemistry and Morphoproteomics

The use of bright field microscopy and immunohistochemistry directed against various protein analytes can better defne the biology of a lymphoma process and the pathogenetic occurrences that might be responsible for its development, chemoradioresistance, and propensity to recur. That is the application of morphoproteomics[9]. To that end, we applied immunohistochemical probes against the following protein analytes in unstained sections of the patient's original diagnostic biopsy read as Hodgkin's lymphoma: CD30 (Dakocytomation Inc., Carpinteria, CA); total insulin-like growth factor (IGF)-1 receptor (R), at tyrosine 1165/1166 (GenWay Biotech Inc. San Diego, CA); mammalian target of rapamycin (mTOR), phosphorylated on serine 2448 (Cell Signaling Technology Inc., Danvers, MA); Akt, phosphorylated on serine 473 (Cell Signaling Technology Inc); signal transducer and activator of transcription (STAT)3, phosphorylated on tyrosine 705 (Santa Cruz Biotechnology, Inc., Santa Cruz, CA); silent mating type information regulation 2 homolog (SIRT)1(Delta Biolabs, Gilroy, CA and Abcam Inc, Cambridge, MA); CD8 (cytotoxic effector T lymphocytes; Dakocytomation Inc.); TIA-1 (T-cell intracellular antigen; cytotoxic granule-associated RNA binding protein (Abcam, Inc., Cambridge, MA); and Foxp3, T regulatory cell marker (Abcam, Inc). The level of expression of the analytes was graded on a 0 to 3+ scale based on signal intensity indicated by a 3,3'-diaminobenzidine tetrahydrochloride (DAB) chromogenic (brown) signal. CD8+, TIA-1+ and Foxp3+ were quantified as relative numbers per high power field.

### Biomedical Analytics

Biomedical analytics develops and applies methods from mathematics and computer science to gain insights into biological processes based on personalized data and published biomedical research. In this study, biomedical analytics integrated the morphoproteomic analysis of a patient's HL with the known effects and interactions of vorinostat (SAHA; suberolyanilide hydroxamic acid) and sirolimus (rapamycin) on signal transduction pathways and immune modulation. The patient's data were normalized and weighted by an algorithm customized for the pathologist of record. The resulting score for each analyte was entered, along with its UNIPROT ID, into Ingenuity Pathway Analysis (IPA, www.ingenuity.com). Pathway networks and their interactions with the proposed therapies were evoked based on existing IPA data. From these graphs and additional data mining of the National Library of Medicine's MEDLINE data base, a single network model was constructed using IPA Pathway Designer to represent the key immune modulation and adaptive responses in the signal transduction processes.
